# Glycocalyx kinetics and injury during liver procurement and transplantation as predictors of early graft dysfunction

**DOI:** 10.3389/frtra.2025.1662187

**Published:** 2025-11-25

**Authors:** Nassiba Beghdadi, Alexis Texier, Marc Antoine Allard, Mylene Sebagh, Nour Bousaleh, Haitham Triki, Daniel Pietrasz, Nicolas Cabrit, Nicolas Golse, René Adam, Cyrille Feray, Peter J. Lenting, Stéphanie Roullet, Daniel Azoulay

**Affiliations:** 1 Hopital Paul Brousse, Villejuif, France; 2 INSERM UMR 1193, Villejuif, France; 3 INSERM UMR-S1176, Le Kremlin-Bicêtre, France; 4 AP-HP Hôpital Paul-Brousse, Laboratoire d'Anatomopathologie, Villejuif, France; 5 Université Paris-Saclay, Villejuif, France

**Keywords:** glycocalyx (MeSH: d019276), ischemia–reperfusion (I/R) injury, liver transplantation complications, syndecan-1 (SDC1), heparan sulfate (HS), angiopoietin (Ang)

## Abstract

**Introduction:**

Ischemia–reperfusion injury causes endothelial damage, partly through degradation of the glycocalyx. This study aimed to evaluate glycocalyx degradation from graft procurement to reperfusion and assess its potential as a biomarker of early graft function (early allograft dysfunction, EAD).

**Methods:**

This single-center observational prospective study was conducted at Paul Brousse Hospital from April 2022 to April 2023. All primary liver transplantation (LT) recipients were included. Glycocalyx degradation was assessed at procurement, at the end of cold ischemia, and during LT in liver graft caval effluent and correlated with liver histological injury. The primary endpoint was EAD, defined as a Model for Early Allograft Function score ≥9. We quantified glycocalyx components [Syndecan-1 (Synd-1), heparan sulfate, angiopoietin-1, and angiopoietin-2], inflammation (TNF-alpha), and cell death markers.

**Results:**

Thirty-one patients were included; 12 (39%) developed EAD. Synd-1 plasma levels at procurement (donor Synd-1 level = d-Synd-1) were significantly higher in patients with EAD [12,173 pg/mL (10,538–17,570) vs. 6,282 pg/mL (4,604–10,002), *p* = 0.004]. A plasma d-Synd-1 cutoff of 9,419.7 pg/mL predicted EAD [AUC = 0.81, 95% confidence interval (95% CI) (0.65–0.97); sensitivity 83%; specificity 74%, positive predictive value = 67%, negative predictive value = 88%, *p* < 0.05]. d-Synd-1 ≥9,419.7 pg/mL was associated with severe post-LT complications (*p* = 0.007).

**Conclusions:**

d-Synd-1 levels in graft effluent during procurement may serve as a predictor of early allograft dysfunction. Strategies aimed at protecting the endothelial during procurement could improve graft outcomes.

## Introduction

Liver graft ischemia–reperfusion injury (IRI) results from the lack of graft oxygenation followed by re-oxygenation and triggers a metabolic cascade leading to cell death and clinical liver dysfunction. Early allograft dysfunction is a clinical expression of IRI. The Model for Early Allograft Function (MEAF) score, a continuous score, is particularly useful for defining severe graft dysfunction (MEAF score ≥9), which corresponds to a 30% risk of graft loss and a 40% risk of patient death within 3 months (1).

The glycocalyx is a glycoprotein-rich membrane that covers the organ endothelium and protects it from circulating blood elements like inflammatory cells (2–7). Syndecan-1 (synd-1) and heparan sulfate (HS) are key components of the glycocalyx and among the most extensively studied components in the literature (2–7). Their structure and function are regulated by blood flow, shear stress, and growth factors like angiopoietins (Ang) (8–12). Shedding of the endothelial glycocalyx occurs when blood circulation ceases, exposing the endothelium to inflammatory cells (13). Increased plasma levels of Synd-1 and heparan sulfate indicate glycocalyx degradation (14–17). Whereas these processes have been studied in cardiac surgery (15, 16) and severe trauma (18, 19), little is known in the context of solid organ transplantation. The aim of our study was to evaluate glycocalyx degradation and its excretion kinetics during graft procurement and to assess whether glycocalyx dynamics could serve as a predictive biomarker of early liver graft dysfunction.

## Materials and methods

### Patients

All consecutive liver transplantations (LTs) performed at Paul Brousse University Hospital (Villejuif, France) between April 2022 and April 2023 were included in the study. Inclusion criteria were patients aged ≥18 years with end-stage liver disease. Exclusion criteria included LT from living donors, split liver grafts, re-transplantation, LT for fulminant hepatitis, use of venovenous bypass during LT, and lack of consent for the use of clinical data. This prospective observational exploratory study was approved by the local Ethics Committee (CO 16-006; CNIL No. 1856085).

### Organ procurement

After procurement, liver grafts were preserved in Institut Georges Lopez-1 (IGL-1®) solution at 4°C until transplantation. Before implantation, all grafts were flushed with 800 mL of 5% albumin solution, according to the protocol of our institution. Total ischemia time was defined as the interval between cessation of blood supply at the time of liver graft procurement and portal reperfusion upon implantation in the recipient. The balance of risk (BAR) score is a composite measure that includes the MELD score, recipient age, donor type (living donor), emergency status of LT, re-transplantation status, and cold ischemia time. It is used to estimate prognosis and survival in liver transplant recipients. A BAR score <9 predicts an excellent prognosis, whereas a score ≥18 indicates a poor prognosis with increased mortality at 3 months post-LT (20). An extended criteria donor (ECD) was defined as a liver graft with macrovacuolar steatosis ≥30%, donor age ≥70 years, elevated GGT, or from a donation after circulatory death (DCD) donor. No liver grafts were perfused using oxygenated dynamic perfusion during the study period.

### Surgical procedure

The retrohepatic inferior vena cava was preserved whenever possible. At our institution, a temporary porto-caval shunt is systematically performed during liver explantation to prevent mesenteric congestion. During graft implantation, a vena cava piggyback anastomosis was created on the hepatic veins. Other anastomoses, including porto-portal, arterial, and biliary or bilio-jejunal, were performed based on the usual practice of the surgical team. Vascular unclamping (caval and portal) was performed simultaneously, followed by arterial unclamping. Abdominal drainage was systematic. Immunosuppression was initiated at the time of surgical incision with methylprednisolone 5 mg/kg (maximum 500 mg). Anesthetic management was at the discretion of the anesthesiologist.

### Study objective

The objective was to assess glycocalyx alteration during liver graft procurement and liver transplantation as a marker of ischemia–reperfusion injury and to determine whether glycocalyx alteration is predictive of early liver graft dysfunction.

The primary endpoint was early allograft dysfunction (EAD), which was evaluated based on the MEAF score. The MEAF is a continuous score calculated from ALT, INR, and bilirubin levels measured on postoperative day 3. It is calculated as follows: MEAF = score ALT_max3POD_ + score INR_max3POD_ + score bilirubine_3POD_, where score ALT_max3POD_ = 3.29/(1 + e^−1.9132^(ln (ALT_max3POD_)) − 6.1723); score INR_max3POD_ = 3.29/(1 + e^−6.8204^(ln (score INR_max3POD_)) − 0.6658); score bilirubine_3POD_ = 3.4/ (1 + e^−1.8005^(ln(bilirubine_3POD_)) − 1.0607). Severe graft dysfunction is defined as a MEAF score ≥9, which corresponds to a 30% risk of graft loss and a 40% risk of patient death within 3 months (1).

The secondary end points were post-LT outcomes: (1) primary non-function corresponding to graft non-function within 7 days post-LT without vascular cause, leading to death or urgent re-transplantation (21); (2) severe post-LT complications defined according to Dindo–Clavien classification as type III, which include severe complications requiring endoscopic, radiological, or surgical intervention (22).

### Experimental protocol

During liver graft procurement, the liver was flushed with IGL-1 solution after the aorta was clamped, ensuring liver graft preservation without blood. To assess endothelial glycocalyx degradation and its excretion kinetics, samples of liver vena cava effluent (blood or preservation fluid/albumin) were collected at specific time points, listed as follows: (i) during liver graft procurement before aortic clamping, (ii) at the end of cold ischemia preservation, after the graft had been flushed with 5% albumin solution through the portal vein, and (iii) 5 min after liver graft reperfusion in the recipient, i.e., after caval and portal unclamping.

### Sample treatment

Blood samples were collected in citrated or EDTA tubes. Blood was centrifuged at 2,000*g* for 10 min to obtain plasma. Plasma samples were aliquoted in 0.5 mL Eppendorf tubes and stored at −80 °C until further analysis by enzyme-linked immunosorbent assay (ELISA). To assess endothelial glycocalyx alteration, we quantified the following biomarkers in the collected samples using ELISA assay kits: Syndecan-1 (human CD138; SDC1, #EHSDC1, Thermo Fisher Scientific, USA), heparan sulfate (E-EL-H2364c, Elabscience Biotechnology Co., France), angiopoietin-1 (EPX010-12235-901, Thermo Fisher Scientific), and angiopoietin-2 (KHC1641, Thermo Fisher Scientific). Ischemia–reperfusion injury was evaluated using inflammation and cell damage markers. Inflammation was assessed by quantifying TNF-alpha (BMS223-4, Thermo Fisher Scientific). Cell death was evaluated by quantifying total cytokeratin 18 (CK-18), a fragment released into circulation upon cell death, reflecting the overall cell death (M65 EpiDeath ELISA, PEVIVA, Eurobio Scientific, France). Cleaved cytokeratin 18 (c-CK-18) is a component generated by enzymatic digestion during apoptosis, enabling the assessment of programmed cell death (M30 Apoptosense Chronic Liver Human, PEVIVA, Eurobio Scientific). Necrosis was indirectly evaluated by subtracting cleaved CK-18 from total CK-18.

### Histology

To evaluate ischemia–reperfusion injury, surgical liver biopsies were performed (1) before aortic clamping in the donor and (2) at the end of LT in the recipient (before abdominal closure). Hepatic tissue damage was assessed by a pathologist, who evaluated neutrophil infiltration, hepatocyte ballooning, and necrosis and classified the ischemia–reperfusion injury as severe, moderate, mild, or absent. We determined whether there was a correlation between histological lesions and excretion of synd-1 or heparan sulfate during liver graft procurement. Histological evaluation was performed blinded to glycocalyx measurements.

### Clinical evaluation

As part of routine clinical care, all patients underwent daily blood tests. Clinical data were collected before, during, and up to 90 days post-transplantation. Clinical, biological, and histological data were collected prospectively.

### Statistical analyses

The study was designed as a prospective, single-center, observational, and exploratory study. Data not normally distributed were presented as median with interquartile range (IQR: 25%–75%). Univariate analyses were performed: chi-square tests were used for categorical variables, and Kruskal–Wallis tests were used for continuous variables. *T*-tests or Mann–Whitney tests were used for multiple comparisons across time points. To assess whether each biomarker was associated with postoperative graft dysfunction, receiver operating characteristic (ROC) curve analyses were performed, including the calculation of positive predictive value (PPV), negative predictive value (NPV), sensitivity (Sen), and specificity (Spe). For all analyses, a *p*-value ≤ 0.05 was considered statistically significant; adjusted *p-*values are reported when data were corrected for multiple comparisons. All statistical analyses were performed using R software (version 2022, Posit Software, PBC).

## Results

### Study population

Between April 2022 and April 2023, 31 patients were included. The median age of the recipients was 55 years (48–63). The MELD score was 16 (8–26) (Supplementary Table S1).

The median age of the donors was 67 years (52–74). The median BAR score was 7 (3–11), and 24 out of 31 donors (77%) met the criteria for ECDs (Supplementary Table S1).

### Kinetics of biomarkers over time

Between liver graft procurement and reperfusion, Synd-1 levels increased significantly [9,035 (5,140–11,774) to 74,577 (55,282–107,969) pg/mL, *p* < 0.001]. Heparan sulfate and Ang-1 decreased significantly (heparan sulfate: 806 (453–1,056) vs. 383 (209–844) ng/mL, *p* < 0.01; Ang-1: 2,732 (992–6,697) vs. 1.361 (651–3,705) pg/mL, *p* < 0.01). Ang-2 level increased, but the change did not reach statistical significance [5,177 (3,676–8,124) vs. 6,669 (3,731–9,281) pg/mL] (Table 1).

**Table 1:** Descriptive analysis of Glycocalyx alteration and cell injuries during liver graft procurement.

Biomarker	Donor sample	End of cold ischemia sample	Reperfusion sample
Syndecan-1 (pg/mL)	9,035 (5,140–11,774)	39,120 (21,395–45,258)	74,577 (55,282–107,969)
Heparan sulfate (ng/mL)	806 (453–1,056)	0 (0–8.9)	383 (209–844)
Angiopoietin-1 (pg/mL)	2,732 (992–6,697)	0 (0–865.2)	1,361 (651–3,705)
Angiopoietin-2 (pg/mL)	5,177 (3,676–8,124)	2,257 (1,029–3,549)	6,669 (3,731–9,281)
Total CK-18 (UI/mL)	1,088 (402–2,188)	124 (68–522)	2,689 (1,697–4,048)
Cleaved CK-18 (UI/mL)	165 (98–229)	236 (130–440)	248 (192–347)
Total−cleaved CK-18 (UI/mL)	1,088 (251–1,868)	0 (0–261)	2,342 (1,616–3,591)
TNF-alpha (pg/mL)	0.16 (0.00–0.94)	0.65 (0.03–2.90)	0.20 (0.00–1.65)

Values are expressed as median (IQR). Total CK-18, total death cell; cleaved CK-18, apoptosis; total−cleaved CK-18, necrosis.

**Figure 1:**
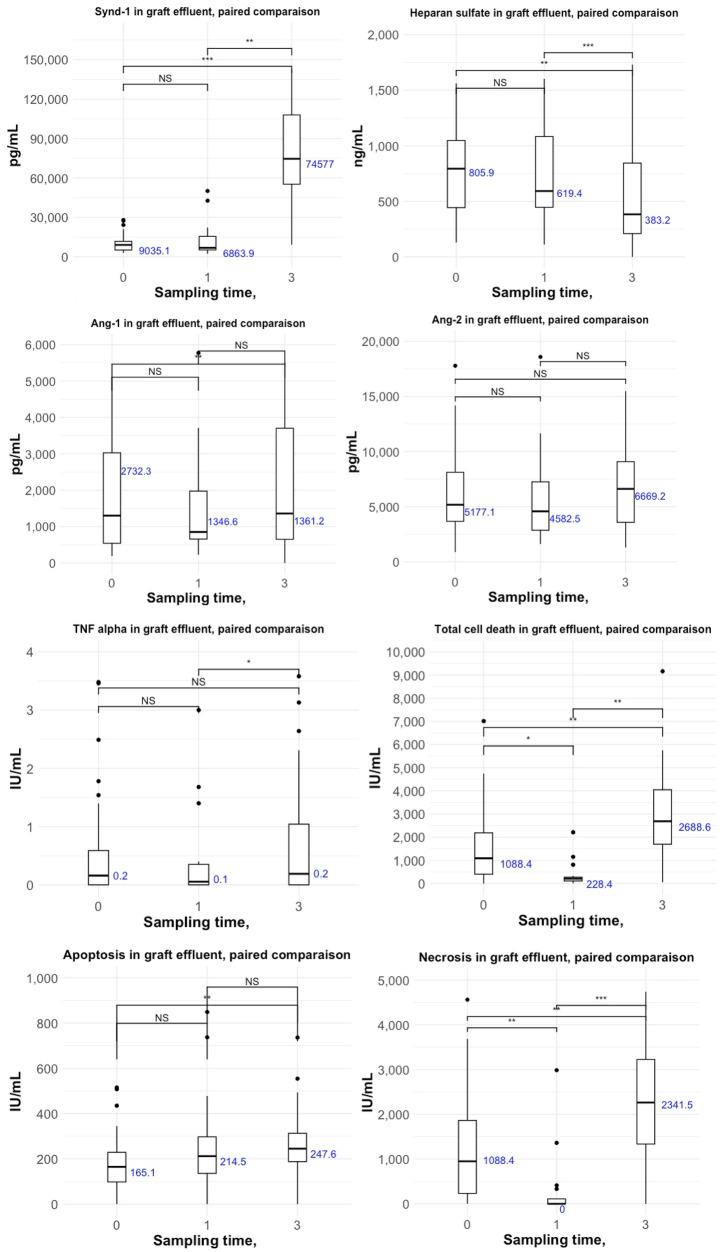
Kinetic evolution of glycocalyx alteration and cell injury during liver graft procurement and transplantation at three time points: (1) donor sampling time, (2) end of cold ischemia, and (3) reperfusion (results are expressed as median and IQR).

Regarding cell injury biomarkers, levels of total CK-18 [2,689 (1,697–4,048) vs. 1,088 (402–2,188) UI/mLl, *p* < 0.01], cleaved CK-18 [248 (192–347) vs. 165 (98–229) UI/mL, *p* < 0.01], and necrosis [2,342 (1,616–3,591) vs. 1,088 (251–1,868) UI/mL, *p* < 0.01] were significantly higher at reperfusion compared to the procurement time point. TNF-alpha levels remained stable in the graft vena cava effluent (Figure 1).

### Early allograft dysfunction according to MEAF scores

**Figure 2:**
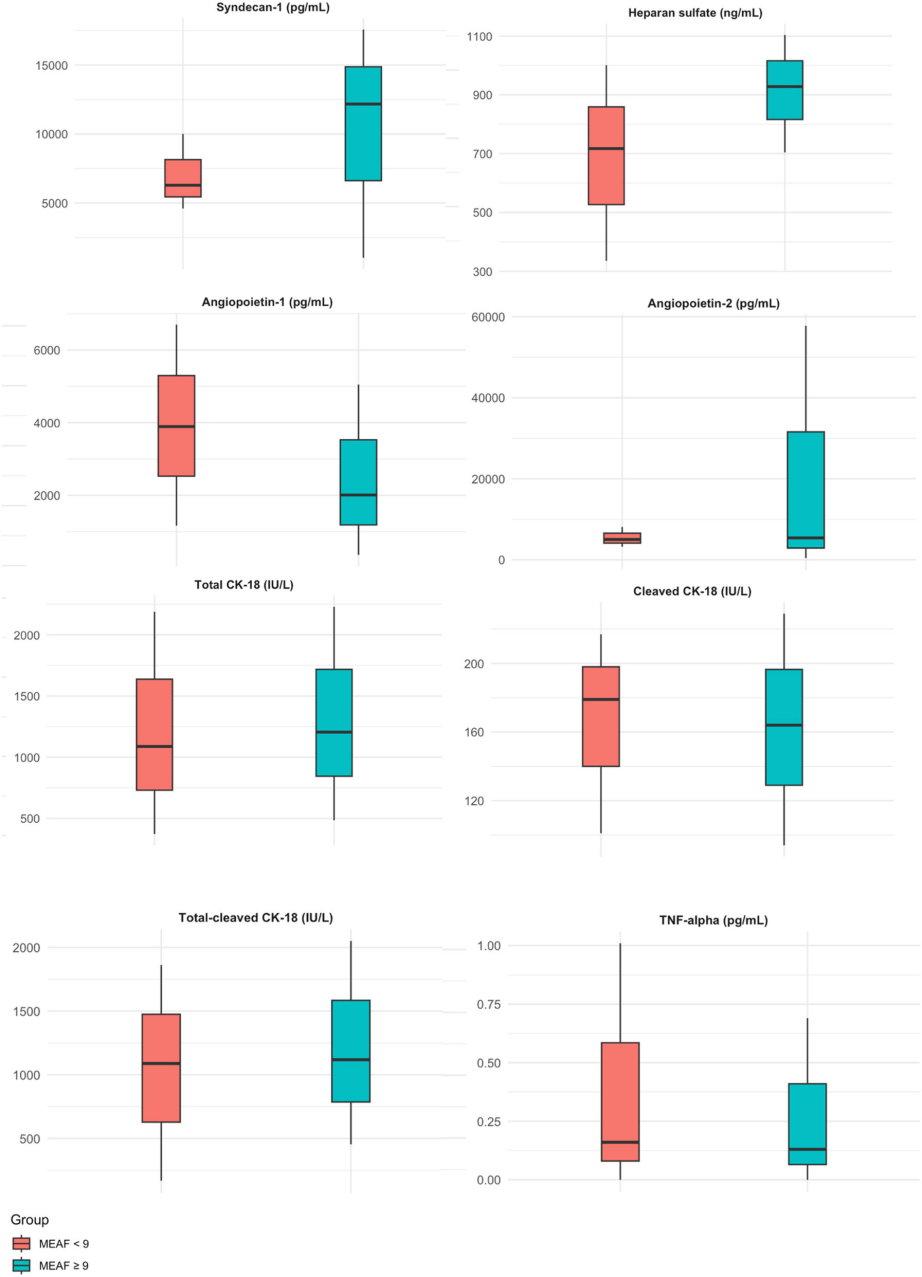
Kinetics of ischemia–reperfusion biomarkers at the time of liver graft procurement according to post-LT graft dysfunction (results expressed as median and IQR).

Twelve patients (39%) developed severe graft dysfunction. Among these, 67% received a graft from a donor who died of anoxia (*p* = 0.001), and they exhibited significant cytolysis [ALT: 117 (22–166) vs. 27 (16–40) IU/L, *p* = 0.019]. No significant differences were observed between patients who developed dysfunction and those who did not regarding recipient age, MELD score, or BAR score (Table 2). Only Synd-1 levels in the graft vena cava effluent at the time of procurement were significantly higher in patients who developed dysfunction: [12,173 (10,538–17,570) vs. 6,282 (4,604–10,002) pg/mL, *p* = 0.004]. These patients also exhibited significantly higher necrosis levels in the graft vena cava effluent at the end of cold ischemia [1,382 (18–2,622) vs. 0 (0–0) IU/L, *p* = 0.004] (Table 3, Figure 2).

**Table 2:** Univariate comparison for early allograft dysfunction MEAF ≥9.

Variable	MEAF < 9 *N* = 19^a^	MEAF ≥9 *N* = 12^a^	*p*-value
Recipient age (years)	55 (48–63)	58 (50–65)	0.655
Male	13 (68%)	8 (67%)	0.919
MELD score	11 (8–32)	17 (13–20)	0.405
Etiology of cirrhosis			
Alcoholic	11 (58%)	3 (25%)	0.073
Viral	2 (11%)	4 (33%)	0.117
Biliary	1 (5.3%)	1 (8.3%)	0.735
MASLD	4 (21%)	4 (33%)	0.447
HCC	4 (21%)	7 (58%)	0.035
Other	5 (26%)	2 (17%)	0.531
Donor characteristics			
Donor age (years)	73 (58–76)	63 (51–74)	0.291
Cause of donor death			
Vascular	13 (68%)	3 (25%)	0.018
Anoxia	2 (11%)	8 (67%)	0.001
Trauma	4 (21%)	1 (8.3%)	0.348
Biology at procurement			
ASAT (IU/L)	42 (24–72)	74 (44–186)	0.071
ALAT (IU/L)	27 (16–40)	117 (22–166)	0.019
*γ*GT (IU/L)	39 (18–86)	44 (34–83)	0.247
Bilirubin (μmol/L)	8.0 (5.7–12.0)	8.5 (5.8–15.0)	0.772
Presence of graft steatosis	13 (76%)	7 (64%)	0.463
BAR score	4.0 (3.0–12.0)	7.0 (3.5–10.0)	0.935
Extended criteria donor	16 (84%)	8 (67%)	0.255
Total ischemia time (min)	332 (302–395)	337 (308–348)	0.921
Graft reperfusion syndrome	13 (68%)	9 (75%)	0.694
Graft ischemia–reperfusion histology			
Severe	1 (5.3%)	1 (8.3%)	0.735
Moderate	9 (47%)	5 (42%)	0.756
Mild	3 (16%)	5 (42%)	0.109
No lesion	4 (21%)	0 (0%)	0.089
Post-LT complications			
PNF	0 (0%)	0 (0%)	
Dindo–Clavien classification			0.661
1	3 (16%)	3 (25%)	
2	9 (47%)	3 (25%)	
3b	1 (5.3%)	1 (8.3%)	
4a	6 (32%)	5 (42%)	
CCI score	42 (22–55)	39 (18–47)	0.683
Dindo–Clavien classification ≥3b	7 (37%)	6 (50%)	0.470
CCI ≥26.6	13 (68%)	8 (67%)	0.919
Mortality	0 (0%)	0 (0%)	
Kidney failure (KDIGO classification)			0.327
0	8 (42%)	9 (75%)	
1	6 (32%)	2 (17%)	
2	1 (5.3%)	0 (0%)	
3	4 (21%)	1 (8.3%)	

a Values are expressed as *n* (%) or median (IQR).

**Table 3:** Univariate analysis according to the MEAF score of the kinetic variation of glycocalyx alteration and cell injury during liver graft procurement and transplantation.

Biomarker	MEAF < 9 *N* = 19^a^	MEAF ≥9 *N* = 12^a^	*p*-value
Donor sample			
Syndecan-1 (pg/mL)	6,282 (4,604–10,002)	12,173 (10.53–17,570)	0.004
Heparan sulfate (ng/mL)	717 (336–1,001)	928 (704–1,104)	0.232
Angiopoietin-1 (pg/mL)	3,892 (1,168–6,697)	2,011 (365–5,047)	0.269
Angiopoietin-2 (pg/mL)	5,009 (3,225–8,124)	5,403 (405–5.7763)	0.704
Total CK-18 (UI/mL)	1,088 (373–2,188)	1,205 (485–2,230)	0.589
Cleaved CK-18 (UI/mL)	179 (101–217)	164 (94–229)	0.761
Total−cleaved CK-18 (UI/mL)	1,088 (168–1,862)	1,118 (454–2,050)	0.503
TNF-alpha (pg/mL)	0.16 (0.00–1.01)	0.13 (0.00–0.69)	0.850
End cold ischemia sample			
Syndecan-1 (pg/mL)	40,988 (38,933–46,212)	19,791 (18,051–38,163)	0.093
Heparan sulfate (ng/mL)	0 (0–10.3)	0 (0–2)	0.328
Angiopoietin-1 (pg/mL)	0 (0–837.2)	0 (0–865.2)	0.511
Angiopoietin-2 (pg/mL)	2,513 (1,415–3,683)	1,468 (1,023–2,605)	0.408
Total CK-18 (UI/mL)	93 (63–176)	1,522 (188–2,921)	0.029
Cleaved CK-18 (UI/mL)	231 (124–452)	236 (148–405)	0.944
Total−cleaved CK-18 (UI/mL)	0 (0–0)	1,382 (18–2,622)	0.004
TNF-alpha (pg/mL)	1.49 (0.28–2.93)	0.03 (0.00–1.62)	0.122
Reperfusion sample			
Syndecan-1 (pg/mL)	78,682 (58,522–1,11,092)	65,858 (48,606–97,542)	0.570
Heparan sulfate (ng/mL)	327 (176–789)	446 (234–829)	0.783
Angiopoietin-1 (pg/mL)	1,611 (890–3,786)	1,128 (639–2,410)	0.521
Angiopoietin-2 (pg/mL)	6,563 (3,833–9,390)	8,068 (3,434–8,845)	0.982
Total CK-18 (UI/mL)	2,689 (1,847–4,038)	2,766 (1,543–4,492)	>0.999
Cleaved CK-18 (UI/mL)	268 (216–410)	221 (180–266)	0.308
Total−cleaved CK-18 (UI/mL)	2,342 (1,700–3,583)	2,562 (1,349–4,272)	0.839
TNF-alpha (pg/mL)	0.45 (0.19–1.93)	0.00 (0.00–0.06)	0.012

a Values are expressed as median (IQR).

Total CK-18, total death cell; cleaved CK-18, Apoptosis; total−cleaved CK-18, necrosis.

HS, Ang-1, and Ang-2 levels did not differ depending on the presence or absence of dysfunction.

Only a few variables differed significantly between the two groups (severe dysfunction vs. non-severe dysfunction), limiting the relevance of a multivariate analysis.

**Figure 3:**
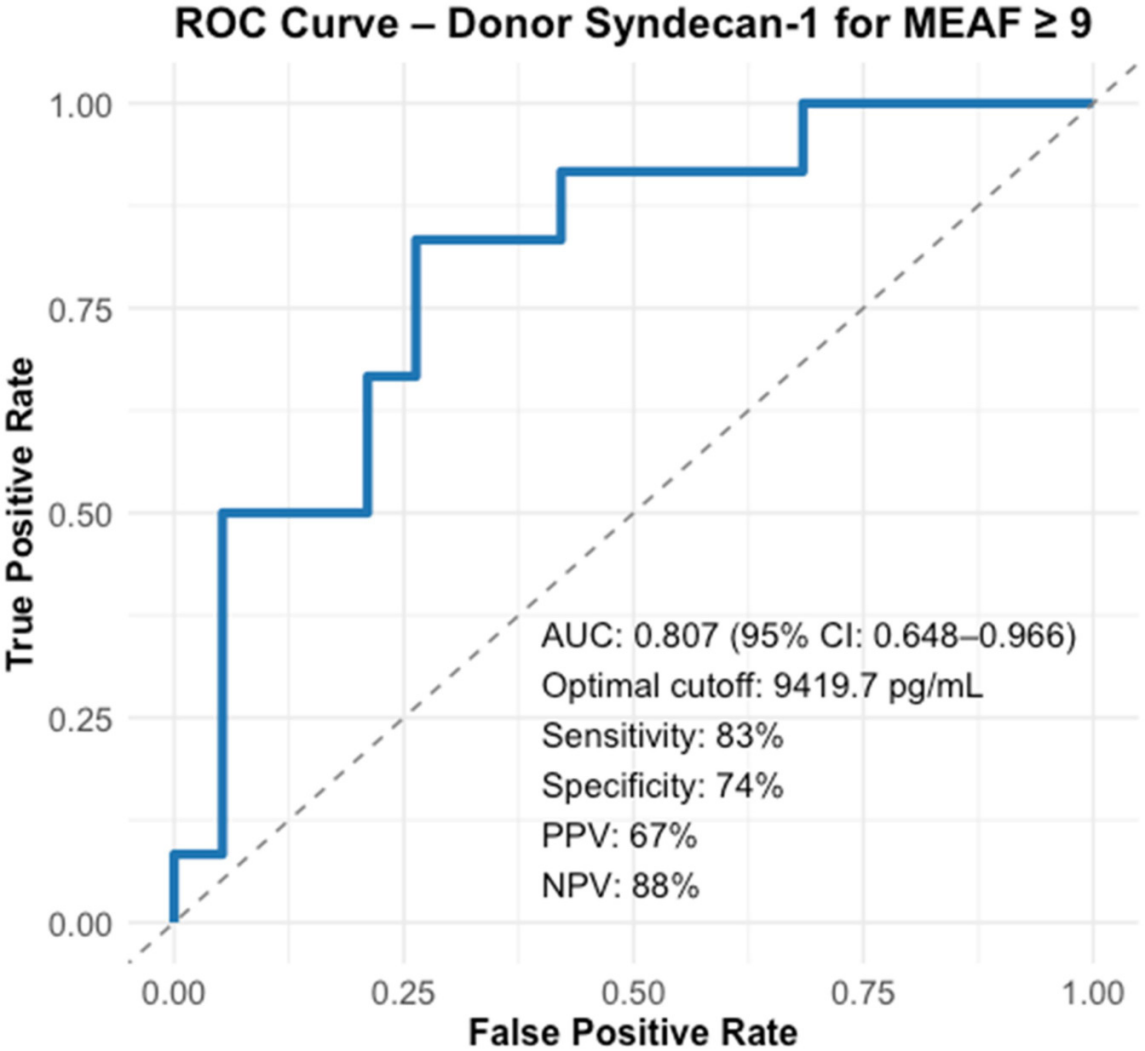
ROC curve identifying the optimal syndecan-1 threshold at the time of donor sampling for MEAF ≥9 (Youden’s test).

The Synd-1 threshold predicting dysfunction was 9.419.7 pg/mL [AUC = 0.81, IC 95% (0.65–0.96), sensitivity = 83%, specificity = 74%, PPV = 0.67, NPV = 0.88, *p* < 0.05] (Figure 3). Liver grafts with Synd-1 ≥9.419.7 pg/mL at the time of procurement exhibited significantly higher cytolysis Aspartate Aminotransférase (ASAT) 77 (56–147) vs. 26 (21–52) IU/L *p* < 0.001; Alanine Aminotransférase (ALAT) 58 (32–150) vs. 20 (12–40) IU/L *p* = 0.004] and cholestasis [GGT 52 (34–148) vs. 28 (15–52) IU/L, *p* = 0.013]. No significant associations were observed between glycocalyx degradation and either blood loss volume or blood product transfusion during LT (Table 4and Supplementary Table S2).

**Table 4:** Clinical characteristics by donor Synd-1 cutoff measured in the vena cava effluent (threshold 9,419.7 pg/mL).

Variable	Synd-1 < 9,419.7 pg/mL *N* = 16^a^	Synd-1 ≥ 9,419.7 pg/mL *N* = 15^a^	*p*-value
Recipient age (years)	57 (45–63)	54 (50–64)	0.8
Male	10 (62%)	11 (73%)	0.7
MELD score	11 (8–18)	17 (11–26)	0.3
Etiology of cirrhosis			
Alcoholic	9 (56%)	5 (33%)	0.2
Viral	2 (12%)	4 (27%)	0.4
HCC	5 (31%)	6 (40%)	0.6
Biliary	1 (6.2%)	1 (6.7%)	>0.9
MASLD	3 (19%)	5 (33%)	0.4
Other	3 (19%)	4 (27%)	0.7
Donor characteristics			
Donor age (years)	73 (58–76)	60 (52–70)	0.089
Cause of donor death			
Vascular	11 (69%)	5 (33%)	0.049
Anoxia	2 (12%)	8 (53%)	0.023
Trauma	3 (19%)	2 (13%)	>0.9
Biology at procurement			
ASAT (IU/L)	26 (21–52)	77 (56–147)	<0.001
ALAT (IU/L)	20 (12–40)	58 (32–150)	0.004
γGT (IU/L)	28 (15–52)	52 (34–148)	0.013
Bilirubin (μmol/L)	8.2 (5.8–14.2)	7.0 (5.5–14.0)	>0.9
BAR score	4.0 (3.0–9.8)	7.0 (3.5–11.0)	0.5
Extended criteria donor	14 (88%)	10 (67%)	0.2
Total ischemia time (min)	325 (292–400)	345 (320–370)	0.6
Graft reperfusion syndrome	10 (62%)	12 (80%)	0.4
Graft ischemia–reperfusion histology			
Severe	1 (6.2%)	1 (6.7%)	>0.9
Moderate	9 (56%)	5 (33%)	0.2
Mild	4 (25%)	4 (27%)	>0.9
No lesion	0 (0%)	4 (27%)	0.043
Post-LT complications			
Mortality	0	0	
Dindo–Clavien classification			0.016
1	1	3 (19%)	
2	2	10 (62%)	
3b	3b	0 (0%)	
4a	4a	3 (19%)	
CCI score	36 (21–47)	47 (36–58)	0.2
Dindo–Clavien classification ≥3b	3 (19%)	10 (67%)	0.007
CCI score ≥26.6	9 (56%)	12 (80%)	0.3
Kidney failure (KDIGO classification)			0.15
0	9 (56%)	8 (53%)	
1	2 (12%)	6 (40%)	
2	1 (6.2%)	0 (0%)	
3	4 (25%)	1 (6.7%)	

a Values are expressed as *n* (%) or median (IQR).

### Biomarkers and postoperative complications

Since only Synd-1 was elevated, we assessed the occurrence of complications beyond the cut off identified on the ROC curve. Recipients who received liver graft with Synd-1 level ≥9,419.7 pg/mL in the vena cava effluent at the time of procurement experienced significantly more severe post-LT complications according to the Dindo-Clavien classification (67% vs. 19%, *p* = 0.007) (TABLE 4).

### Biomarkers of glycocalyx alterations and histological ischemia–reperfusion injuries in liver biopsies.

Despite elevated Synd-1 levels, liver grafts did not show evidence of severe histological injury after graft reperfusion (Table 4).

## Discussion

Our study is the first to investigate glycocalyx alterations and angiopoietin secretion during liver transplantation. Synd-1 levels in the cava effluent at the time of procurement appeared to serve as a predictive marker of graft dysfunction.

Schiefer et al. measured Synd-1 levels in the graft cava effluent at the end of cold ischemia (23), reporting a median level of 4.720 ng/mL. In our study, Synd-1 levels in the vena cava effluent at the end of cold ischemia were lower, with a median concentration of 39.12 ng/mL. In the study by Schiefer, the liver grafts were preserved in histidine–tryptophan–ketoglutarate (HTK, Custodiol®) solution, whereas we preserved the organs in IGL-1® solution. IGL-1® has been shown to protect against glycocalyx alteration (24). While Schiefer et al. reported a significant increase in Synd-1 levels in the cava effluent at the end of cold ischemia, we did not find this variation in our study, and Synd-1 levels were similar in patients with graft dysfunction.

Schiefer et al. demonstrated that the glycocalyx is primarily degraded during cold ischemia (23). However, they did not indicate baseline levels of Synd-1 secretion in the graft prior to procurement and after reperfusion. In our study, we observed an upward trend in Synd-1 release within the graft from organ procurement to its reperfusion, suggesting continuous degradation of the glycocalyx within the graft during transplantation.

They highlighted a positive correlation between the Synd-1 release in the vena cava effluent at the end of cold ischemia and biomarkers of liver injury (23). In our study, no peak in postoperative cytolysis was observed; however, patients with very high Synd-1 levels experienced significantly more post-LT kidney dysfunction (data not available) (25).

Passov et al. focused on what happens within the graft during LT and in the peripheral blood circulation of the recipient (26). They collected blood samples from the portal and hepatic veins before and after portal unclamping. Synd-1 levels showed a significant increase from the portal vein to the hepatic veins, while heparan sulfate levels showed a significant decrease; however, these changes did not correlate with graft dysfunction. They hypothesize that the decrease in heparan sulfate in the vena cava effluent reflects its utilization in repairing the glycocalyx of the graft. This decreasing trend in heparan sulfate within the graft is consistent with our results. In kidney transplantation, the glycocalyx thickens within 30 min after the reperfusion of the kidney graft (27). The infusion of exogenous heparan sulfate seems to contribute to the reconstruction of the glycocalyx (28, 29). This finding strengthens the hypothesis that the early decrease in heparan sulfate may be secondary to its role in the early regeneration of the glycocalyx. Another hypothesis could be that Synd-1 is more stable than heparan sulfate in plasma (30).

It is also worth noting that the type of preservation solution plays a role in preventing glycocalyx damage, as Synd-1 levels at the end of cold ischemia in our study were 100 times lower than those reported by Schiefer et al. (23). Contrary to the data in the literature, we found no association between TNF-alpha levels and glycocalyx degradation within the graft (31–33).

Our study is the first to measure variations in Ang-1 and Ang-2 alongside Synd-1 and heparan sulfate. At baseline, Ang-1 is bound to the Tie-2 receptor on the surface of endothelial cells, helping to maintain and stabilize endothelium barrier function by inducing anti-inflammatory and pro-survival intracellular signaling pathways (34). Secretion of Ang-2 by endothelial cells in response to injury destabilizes the endothelium because it competes with Ang-1 for binding to Tie-2 (35). During ischemia, Ang-2 activity increases, while Ang-1 levels in plasma significantly decrease (36, 37). We observed a similar trend in our study, although the changes did not reach statistical significance.

### Limitations

The sample size in our study remains small and might not be robust enough to detect correlations; therefore, the results should be interpreted with caution. In addition, for cost reasons, we only measured one inflammatory cytokine, which showed little variation, contrary to findings described in the literature. Also, sampling was limited to 1 h after portal reperfusion of the graft, preventing analysis of biomarkers in the days following transplantation. The possible impact of medications on the glycocalyx must also be considered; particularly, glucocorticoids, potent anti-inflammatory agents, have been shown to exert protective effects on the endothelium (38, 39).

Finally, we have a liver transplantation with a DCD graft, which may represent a confounding factor in the study due to differences in procurement conditions. It would be useful to perform additional analyses involving a larger number of DCD donors.

### Perspectives

We have shown that measuring glycocalyx degradation markers as early as organ procurement can predict future liver graft function. Such early measurements could provide objective biological support to assist in graft selection. This study highlights the importance of glycocalyx preservation.

## Conclusion

Glycocalyx degradation appears to be an early and significant event in the ischemia–reperfusion injury cascade. Synd-1 may serve as a predictive biomarker of future liver graft function. These findings underscore the importance of considering studies that aim to develop strategies for preserving and protecting the endothelium during liver transplantation.

## Data Availability

The raw data supporting the conclusions of this article will be made available by the authors, without undue reservation.
