# Supplementary material for: Glycocalyx kinetics and injury during liver procurement and transplantation as predictors of early graft dysfunction
Source: Front Transplant. 2025 Nov 25;4:1662187. doi: 10.3389/frtra.2025.1662187 (PMC12687380; doi:10.3389/frtra.2025.1662187)
Supplement: Supplementary file 1 [file Datasheet1.docx]

**Supplementary Table 1 :** Descriptive data

n (%) ; Median (IQR)

| Descriptive data | |
| --- | --- |
|  | N = 31*^1^* |
| Recipient age, year | 55 (48, 63) |
| Male, n (%) | 21 (68%) |
| MELD score, | 16 (8, 26) |
| Etiology of cirrhosis |  |
| Alcoholic, n (%) | 14 (45%) |
| Viral, n (%) | 6 (19%) |
| Biliary, n (%) | 2 (6.5%) |
| MASH, n (%) | 8 (26%) |
| HCC, n (%) | 11 (35%) |
| Other, n (%) | 7 (23%) |
| Donor caracteristics |  |
| Donor age, year | 67 (52, 74) |
| Cause of donor death |  |
| vascular, n (%) | 16 (52%) |
| anoxia, n (%) | 10 (32%) |
| trauma, n (%) | 5 (16%) |
| BAR score | 7.0 (3.0, 11.0) |
| Extended criteria donor, n (%) | 24 (77%) |
| Total ischemia time, min | 334 (306, 382) |
| Graft Ischemia-reperfusion Histology |  |
| Severe, n (%) | 2 (6.5%) |
| Moderate, n (%) | 14 (45%) |
| Mild, n (%) | 8 (26%) |
| No lesion, n (%) | 4 (13%) |
| Post LT Complications |  |
| PNF, n (%) | 0 (0%) |
| MEAF score | 8.83 (8.37, 9.11) |
| MEAF score ≥9, n (%) | 12 (39%) |
| Complications, n (%) | 25 (81%) |
| Calvien-Dindo classification, n (%) |  |
| 1 | 6 (19%) |
| 2 | 12 (39%) |
| 3b | 2 (6.5%) |
| 4a | 11 (35%) |
| CCI score | 42 (21, 50) |
| Calvien-Dindo classification ≥3b | 13 (42%) |
| CCI ≥26.6 | 21 (68%) |
| Mortality, n (%) | 0 (0%) |
| Kidney failure (KDIGO classification), n (%) |  |
| 0 | 17 (55%) |
| 1 | 8 (26%) |
| 2 | 1 (3.2%) |
| 3 | 5 (16%) |

**Supplementary Table 2 :** **Univariate comparison for Early allograft dysfunction MEAF ≥9**

n (%) ; Median (IQR)

|  | MEAF<9  N = 19 | MEAF ≥9  N = 12 | p-value |
| --- | --- | --- | --- |
| Blood product transfusion, n (%) | 12 (63%) | 9 (75%) | 0.492 |
| Blood loss volume, mL | 2 000  (1 250-3 050) | 1 850  (1 350- 5 050) | 0.761 |
| Blood loss volume >2 000mL,  n (%) | 10 (53%) | 6 (50%) | 0.886 |
| Total number of RBC  units transfused, n | 2.0 (0.0- 5.5) | 3.5  (0.0- 4.2) | >0.999 |
| Recipient Mean arterial pressure at graft reperfusion, mmHg | 82 (72-87) | 73 (66-82) | 0.263 |
| Norepinephrine dose at graft reperfusion, mg/h | 2.5  (0.6-4.0) | 4.8  (1.8-7.2) | 0.200 |

RBC= red blood cell

|  | Synd 1 <9419.7 pg/mL  N = 16*^1^* | Synd 1 ≥9419.7 pg/mL  N = 15*^1^* | p-value |
| --- | --- | --- | --- |
| Blood loss volume, mL | 1 500 (1 125- 2 375) | 3 000 (850-5100) | 0.7 |
| Total number of RBC  units transfused, n | 2.0 (0.0-5.0) | 4.0 (0.0-4.5) | 0.9 |
